# Editorial: Sustainable and Environmentally Concerned Citizens: Garden-Based Learning to Promote the Importance of Physical, Natural, and Social Resources

**DOI:** 10.3389/fpsyg.2021.703057

**Published:** 2021-08-05

**Authors:** Raquel Perez-Lopez, Marcia Eugenio-Gozalbo, Edward Edgerton, Juan Ignacio Aragones

**Affiliations:** ^1^Department of Research and Psychology in Education, School of Education, Complutense University of Madrid, Madrid, Spain; ^2^Department of Didactics of Experimental, Social, and Mathematical Sciences, Faculty of Education of Soria, University of Valladolid, Soria, Spain; ^3^Psychology Division, School of Education and Social Sciences, University of the West of Scotland, Paisley, United Kingdom; ^4^Department of Social, Organizational and Differential Psychology, School of Psychology, Complutense University of Madrid, Madrid, Spain

**Keywords:** green space, sustainability, environmental concern, environmental education, garden-based learning

We are in the midst of a global environmental crisis, which has been clearly established by scientists, in such a way that the preservation of the planet constitutes the main concern and duty if we truly care not only about future generations, but also about our own health and well-being (Lewis and Maslin, 2015). To these purposes, scientific dissemination and sustainability education constitute fundamental pillars of contemporary citizenship. It is widely recognized that private actions have public implications, and it appears as urgent to search for the common good for all, based on reasons of justice, equality, and equity, and to achieve a sustainable society.

Research evidence demonstrates that contact with nature relates to psychological aspects and these nature-based experiences seem to play an important role in the emotional, cognitive and evaluative development (Kellert, 2002). Concretely, Pirchio et al. (2021) found that, in comparison to participants in the control group, students taking part in outdoor education programs reported positive outcomes in psycho-physical wellbeing, connectedness to nature and pro-social behavior. However, contact with nature is becoming more infrequent in an increasingly urbanized world. Such loss of human-nature interactions was already reported two decades ago and continues to rapidly increase. Among others, it results in threats and damages to health and well-being and causes a disconnection of what supports our lives, i.e., nature.

Therefore, it is important to increase education in nature or to naturalize school environments, for instance by incorporating gardens to school backyards across all educational stages. Instructional gardens constitute versatile tools that are used worldwide to accomplish a wide range of purposes. In fact, Garden-Based Learning (GBL) is an educational methodology based on experiential learning which uses gardens to implement programs and projects from an interdisciplinary approach (Desmond et al., 2002). Thus, students gain knowledge due to the transformation occurring after such real-world experience. School and urban gardens are spaces that facilitate learning processes because they imply active and concrete experiences that help to assimilate information and create abstract concepts. This relatively new approach to education is relevant to children and young people as they are considered more active, “hands-on,” and visual learners. School gardens have been used mainly for three purposes: teaching sciences (Mabie and Baker, 1996; Klemmer et al., 2005), promoting nutrition education (Morgan et al., 2010), and increasing motivation (Ruiz-Gallardo et al., 2013). Recently, the use of school gardens' is becoming more complex, helping to encourage not just environmental awareness or knowledge in students, but other educational aspects such as autonomous learning or personal growth, as it is shown in the present Research Topic.

The primary aim of this Research Topic was to gather evidence on how learning gardens may represent naturalized environments to grow and play, as well as spaces where children and young people might develop more sustainable environmental attitudes along with motivating learning experiences. More specifically, our aim was to improve understanding of how learning gardens might contribute to instruct citizens who are more concerned and caring toward nature, promoting values, knowledge, and behaviors that might help to preserve the environment and help to face the global environmental crisis.

In this issue, a variety of investigations support the importance of using GBL as a tool for stimulating understanding of the natural world across different educational levels. In elementary education, Luís et al. showed how green spaces, namely, schoolyards in primary schools, constitute restorative places. These authors found that children who had less contact with nature benefitted more in schools with earther areas and vegetable gardens apart from trees. Pollin and Retzlaff-Fürst demonstrated that children in sixth grade experienced more positive emotions and social interactions when they take lessons in the school garden than in regular classrooms. From a distinctive point of view, Löfström et al. presents a novel study that introduces nature as a source of disruption instead of a prompt for well-being or happiness. They proposed a methodology based on provocative eco-visualizations (e.g., images of local plastic recycle plants) that trigger emotions in primary education students. Using “Nature in Your Face” methodology as a pilot experience, these authors reported that children in fifth grade might transform their knowledge into actions because they reflected about the way the natural environment has been treated. Finally, Eugenio-Gozalbo et al., used graphical representations of a garden to asses key science topics where learning is promoted at the preschool, primary, secondary, and university stages when gardens are used as instructional resources. Such topics included knowledge on plants at all stages, the establishment of cause-effect relationships (preschool and primary school), water use (most stages), and sustainable techniques of cultivation (University).

Nowadays, the use of GBL in Higher Education has increased and this is reflected in the papers by (Cheang et al., Eugenio-Gozalbo et al., Hurtado-Soler et al.). Therefore, Hurtado-Soler's et al. research revealed that GBL goes beyond the acquisition of knowledge, it might be considered as a holistic and global approach. They found that undergraduate teachers, through sensorial perceptions derived from experiences in different spaces, were able to reflect on their relationships with the environment. Similarly, Cheang et al. conducted research with undergraduates involved in an Ecogarden-based program. These authors evaluated a 5-year program from a phenomenographic approach and concluded that students improved their knowledge and competences, environmental awareness and skills (e.g., personal growth or development of career progress).

The research papers included in this special issue have demonstrated the value of GBL programs by means of a wide range of results, including psychological outcomes such as social competence, emotional behavior, restorativeness, attitudes to nature, connectedness to nature, and personal growth as well socio-educational outcomes such as awareness of environmental issues, increases in knowledge, and skill level and enhancement of career development. Within these results, it is worth highlighting two specific findings: (i) long-term impact of GBL programs on participants' pro-sustainability knowledge, skills and attitudes, and (Cheang et al.) (ii) the restorative impact of GBL programs may be stronger for some groups than for others e.g., children who generally have lesser contact with nature (Luís et al.).

Despite concerns about the internal validity of real-world research such as that conducted in these papers, the findings from these studies suggest that GBL programs can be successfully applied across the educational spectrum from pre-school to Higher Education and have considerable potential for enhancing a broad range of psychological, social, and educational outcomes.

## Author Contributions

All authors listed have made a substantial, direct and intellectual contribution to the work, and approved it for publication.

## Conflict of Interest

The authors declare that the research was conducted in the absence of any commercial or financial relationships that could be construed as a potential conflict of interest.

## Publisher's Note

All claims expressed in this article are solely those of the authors and do not necessarily represent those of their affiliated organizations, or those of the publisher, the editors and the reviewers. Any product that may be evaluated in this article, or claim that may be made by its manufacturer, is not guaranteed or endorsed by the publisher.
